# Model of yearly transition to severe trachomatous scarring and trichiasis in a cohort of women in Kongwa Tanzania

**DOI:** 10.1038/s41598-024-67245-w

**Published:** 2024-07-19

**Authors:** Sheila K. West, Ashley Hazel, Beatriz Munoz, Meraf A. Wolle, Harran Mkocha, Travis C. Porco

**Affiliations:** 1 Dana Center for Preventive Ophthalmology, Wilmer Eye Institute, Johns Hopkins Medicine, Baltimore, MD USA; 2 Proctor Foundation, University of California San Francisco, San Francisco, CA USA; 3 Kongwa Trachoma Project, Kongwa, Tanzania

**Keywords:** Diseases, Medical research, Pathogenesis, Risk factors

## Abstract

One criterion for validation of trachoma elimination is the management of Trachomatous Trichiasis (TT) after Trachoma inflammation—follicular (TF) is eliminated in children ages 1–9 years at district level. No data exist on how long countries must have dedicated TT programs, as the timeline for progression to TT from trachomatous scarring is unknown. We used eight years of longitudinal data in women in Kongwa Tanzania to model progression from no scarring (S0) through grades of scarring severity (S1–S4) to TT. Markov models were used, with age, community prevalence of TF (CPTF), and household characteristics as co-variates. Adjusted for covariates, the incidence of S1 was estimated at 4∙7% per year, and the risk increased by 26% if the CPTF was between 5–10% and by 48% if greater than 10%. The transition from S4 to TT was estimated at 2∙6% per year. Districts, even after elimination of TF, may have some communities with TF ≥ 5% and increased risk of incident scarring. Once scarring progresses to S2, further progression is not dependent on CPTF. These data suggest that, depending on the district level of scarring and degree of heterogeneity in CPTF at the time of elimination, incident TT will still be an issue for decades.

## Introduction

Trachoma, the result of repeated infections with *Chlamydia trachomatis*, is the leading infectious cause of blindness world-wide^1^. The World Health Organization (WHO) has targeted the elimination of trachoma by 2030 using a multipronged strategy including trichiasis surgery^1^. Trachomatous Trichiasis (TT) is the result of several bouts of infection causing scarring (TS) of the conjunctiva and entropion. When severe, entropion can result in trichiasis or rotation inward of the eyelashes until they touch the globe. Trichiasis is the blinding end result of trachoma, and together with scarring, is seen mostly in women^2^. Surgery directs the lashes away from the cornea, preventing vision loss, so country trachoma programs have mounted extensive surgical campaigns to reduce the TT burden^3^.

The WHO validates the elimination of trachoma as a public health problem when three criteria are met: the prevalence of Trachomatous Inflammation—Follicular (TF) in children ages 1–9 years at district level is < 5%; the prevalence of TT unknown to the health system in those ages 15 + is < 2/1000 population; evidence that the health system can continue to identify and manage incident cases of TT (1). After TF is eliminated, TS can continue to progress and produce TT that needs to be managed by the health system^4,5^. While the first two criteria can be met following population-based prevalence surveys that signal the end of a targeted program, the last one requires an open-ended commitment. As more countries reach elimination, the need for data is urgent on how long health ministries must plan for an active TT surgery program in formerly endemic districts.

There are no data on the time course for progression from having no scarring, to scarring, to severe enough scarring to produce TT. This is especially critical in the context where TF is very low, and the uncertainty of incident scarring is an issue. This study used longitudinal data on scarring incidence and progression in women living in a formerly endemic district in Tanzania to model yearly chance of progression, and determine associated personal, household, and community factors that might be associated with progression.

## Methods

All research was reviewed and approved by the Institutional Review Board at Johns Hopkins Medicine, and the Tanzania National Institute for Medical Research. All participants provided informed written consent. All methods and procedures were carried out in accordance with the Declaration of Helsinki.

Images were taken of the everted right eyelid using a Nikon D-40 camera with a 105 mmf/2·8D AF Macro Nikkor Autofocus Lens with a 2.5-inch LCD screen. The images were downloaded and sent to Johns Hopkins, where they were graded by two trained graders for scarring in the upper eyelid using a six-step severity scale as follows^6,7^:

S0: no lines of scarring at least 3 mm in length (less severe than grade S1).

S1 (minimal): one or more lines of scarring at least 3 mm in length but total scarring occupying < 1/8 of the eyelid (not as severe as S2).

S2 (modest): multiple lines or patches of scarring = 1/8 of the eyelid, but total scarring < 1/3 of the eyelid (not as severe as S3A).

S3A (moderate): scarring = 1/3 of the eyelid with clear conjunctiva between, but total scarring < 50% of the eyelid (not as severe as S3B).

S3B (moderately severe): scarring = 50% of the lid, but total scarring occupying ≤ 90% of the eyelid (not as severe as S4).

S4 (very severe), scarring > 90% of the conjunctiva.

Images were assessed by two independent trained graders, graded at 5× magnification on computer screens with at least 1920 × 1080 resolution, and disagreements were openly adjudicated. Graders were masked to all participant data and to each other’s grades.

Trachomatous Trichiasis was determined at the time of the surveys by a trained trachoma grader in the field and defined as at least one eyelash touching the globe, or evidence (documented in an image) of epilation, or history and evidence (surgical scar) of trichiasis surgery. Degree of entropion was not recorded. All TT cases were referred for management and a log created for the community health worker for follow-up.

### Study sample

**Figure 1:**
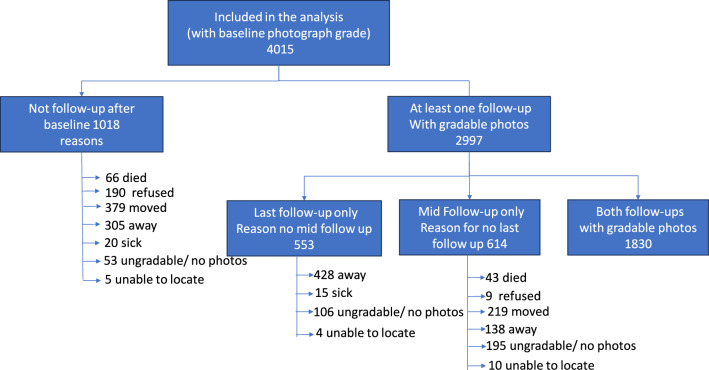
Diagram of participation and loss to follow-up throughout the study.

In 2013, during a randomized trial of 48 communities, we conducted a census and selected a random sample of 100 women aged 15 years and older per community for a study of scarring and cooking fires^8,9^ These women formed the baseline cohort of the current study, and all women with baseline images graded for scarring and who did not have trichiasis were eligible (N = 4015, Fig. 1). In 2016, the first follow-up occurred and 2444 eligible women participated. In 2021, the second follow-up occurred for everyone who was eligible at baseline. Overall, 2997 women were enrolled for two or more visits. From 2013 to 2016, study communities with TF prevalence > 5% received MDA annually, and the entire district was treated by the National Trachoma Program. During the period 2016 to 2021 no annual MDA was provided to the district.

At baseline, data were collected on household characteristics: observations on the construct of the roof (mud or tin); presence of a functional latrine; questions on the educational level of the head of household, ownership of a bicycle or cell phone; and the time (one way) to collect water. We had data on the community prevalence of TF (CPTF) in 1–9 year-olds for the 48 communities at baseline and in 2015. In 2018, as part of a district wide survey, 16 of the 48 villages were included (Supplementary Table 1A,B).

### Definitions

Incident scarring was defined as progression from S0 to S1. Progression of existing scarring was defined as going from a lower grade to higher grade of scarring. We assumed there was no regression of scarring as there is no biological reason scarring would regress. Incident trichiasis was defined as having no trichiasis on a previous visit and developing trichiasis at a subsequent visit.

### Sample size

While our study prespecified the use of a multistate Markov model, we assessed the power using a simplified comparison: the TT incidence for those with S0–S1 versus those with S2–S4. Partly based on our prior data^9^, we assumed the incidence of TT in those with S0–S1 was 0.5 per 100-person years (PYs). Assuming a 75% follow-up rate, we expected data on at least 1700 individuals of whom 12% would have S2–S4 scarring at baseline followed for 8 years. Comparing the TT incidence in those with S2–S4 to that among those with S0–S1, we anticipated over 80% power to detect an incidence rate ratio of no less than 2.4, assuming an alpha of 0.05, and using a two-sided test (based on the arcsine transformation of the binomial test). The planned analysis used the spectrum of scar grades (unlike the simplified power calculation), and we anticipated adequate power for the planned analysis.

### Analytic methods

For univariate and multivariate analyses, CPTF was trichotomized into groups representing elimination (TF < 5%), close to elimination (TF = 5–10%), and greater (TF > 10%).

We fit a continuous-time Markov chain to the progression of scarring. Individuals were assumed to progress from stage 0 through the successive stages through to TT (Supplementary Fig. 1). We used data from the baseline and the two follow-up visits, fitting the model cross-sectionally and thereby assuming interval censoring for transition times (including those to TT). We assumed the transition intensities could be modified by the available covariates. We assumed transitions were possible from each scar stage to the next, as well as from stage S3B and S4 to TT.

We fit univariate models, computing the relative intensity (relative rate) for each covariate and each intensity. We then fit an overall multivariate model. Fitting was conducted using maximum likelihood, with P-values estimated by the likelihood ratio test compared with a model in which the covariate was excluded for each specific intensity. Overall P-values for univariate models were computed by omitting the covariate from all intensities in the model and calculating the likelihood ratio statistic. Based on the fitted continuous time model, we then calculated the yearly probabilities for undergoing transitions from each scar state to the next more severe state (or to TT) based on assuming the mean values for all covariates.

For multivariate models, we report a (full) multivariate model in which all seven transitions are predicted by each of the following covariates: age; time period (first or second); CPTF; presence of a latrine, bicycles, telephone, and mud roof; time to water; education of head of household. We then constructed a more parsimonious model using stepwise regression by (1) eliminating all regressors for any arrow for which the estimated T statistic from the fitted full model was less than 1 and (2) using a forwards-backwards procedure based on the Bayes Information Criterion (with each person-phase counting as one observation). The procedure allowed for covariate effects in different transitions to be constrained to be the same, provided that all such constrained arrows were contiguous in the flow graph of the model.

As a measure of model fit, we calculated the relative McFadden R^2^, defined as the McFadden R^2^ for the chosen model divided by the McFadden R^2^ derived from a saturated model indicating the best possible fit^10^. A saturated model was obtained by stratifying flow rate estimates by starting state, and separately fitting all possible follow-up time periods observed.

All calculations were performed in R v. 4.3.0 for MacIntosh (R Foundation for Statistical Computing, Vienna, Austria), package ‘msm’, and package ‘rolog.’

## Results/findings

Of the 4015 women enrolled at baseline, 67∙5% had a grade of S0, and the mean CPTF was 5∙4% (Table 1). The women who were followed at least once tended to be slightly older and have less severe scarring compared to those lost to follow-up. There were differences between the cohort who missed the first follow-up visit, and those who missed the last follow-up or had both follow-ups (Supplementary Table 1).

**Table 1:** Baseline characteristics by follow-up status.

Baseline characteristics	Followed at least once	Not followed after baseline	All	P-value
N	2997	1018	4015	
Age in years				
Mean (SD)	35.6 (15.6)	32.9 (17.3)	34.9 (16.1)	< 0.0001
Median	32	27	31	
Min–Max	15–94	15–95	15–95	
Community TF prevalence (CPTF)				
Mean (SD)	5.5 (3.8)	5.3 (4.0)	5.4 (3.8)	0.19
Median	4.8	4.2	4.8	
Min–Max	0–15.7	0–15.7	0–15.7	
Years of formal education				
Mean (SD)	3.9 (3.5)	3.7 (3.7)	3.8 (3.5)	0.38
Median	4.0	4.0	4.0	
Min–Max	0–14	0–14	0–14	
Family owns a bicycle (n (%))	1410 (47.3)	424 (41.9)	1834 (45.9)	0.003
Family owns a phone (n (%))	1355 (45.5)	459 (45.3)	1814 (45.4)	0.93
House has a mud roof (n (%))	340 (11.5)	161 (15.9)	501 (12.5)	0.0003
House has a latrine (n (%))	2403 (80.8)	787 (77.8)	3190 (80.0)	0.045
TS grade n (%)				
S0	2073 (69.2)	637 (62.6)	2710 (67.5)	0.014*
S1	409 (13.7)	203 (19.9)	612 (15.2)	
S2	332 (11.1)	95 (9.3)	427 (10.6)	
S3A–S3B	120 (4.0)	63 (6.2)	183 (4.6)	
S4	63 (2.1)	20 (2.0)	83 (2.1)	

*Mantel–Haenzel Test for trend.

Age was associated with the transition between scarring states (Table 2). The second follow-up period, the 4.5-year interval between the two follow-up periods, was associated with a higher rate of scarring transition in some cases, and a lower rate of transition to TT.

**Table 2:** Age-based, time-dependent model for scarring transition. Transition rates are evaluated at the mean covariate levels.

Transition	Transition rate (per year)	Relative rate, age (decade)	Relative rate, second follow-up period
S0 to S1	0.047 (0.042, 0.053)	1.319 (1.26, 1.382)	11.094 (9.007, 13.665)
S1 to S2	0.124 (0.112, 0.138)	1.252 (1.186, 1.321)	1.36 (1.113, 1.663)
S2 to S3A	0.178 (0.158, 0.2)	1.056 (0.997, 1.117)	2.274 (1.84, 2.81)
S3A to S3B	0.489 (0.419, 0.572)	1.065 (0.99, 1.147)	0.761 (0.581, 0.997)
S3B to S4	0.124 (0.098, 0.158)	1.16 (1.057, 1.274)	1.43 (0.966, 2.118)
S3B to TT	0.007 (0.001, 0.061)	1.035 (0.738, 1.452)	0.033 (0, 4.634)
S4 to TT	0.025 (0.011, 0.058)	1.044 (0.791, 1.379)	0.083 (0.021, 0.327)

Akaike Iinformation Criterion (AIC): 7964.794.

CPTF was significantly associated with the transition of scarring from S0 to S1 only (Table 3). Relative to a CPTF of < 5%, women in communities with CPTF of 5–10% had a 30% increased rate of transition to scarring, and those in communities where CPTF > 10% had a 52% increased rate of transition to scarring. No interaction was detected between baseline CPTF rate and the relative rate for the second time-period (data not shown).

**Table 3:** Time-dependent model for scarring transition, including baseline CPTF prevalence categories. Transition rates are evaluated at the mean covariate levels.

Transition	Transition rate (per year)	Relative rate, 5–10% TF prevalence	Relative rate, > 10% TF prevalence	Relative rate, second follow-up period
S0 to S1	0.045 (0.04, 0.05)	1.291 (1.116, 1.495)	1.523 (1.268, 1.828)	11.64 (9.453, 14.33)
S1 to S2	0.132 (0.119, 0.146)	1.075 (0.889, 1.298)	1.229 (0.971, 1.555)	1.436 (1.177, 1.753)
S2 to S3A	0.185 (0.166, 0.206)	1.138 (0.929, 1.394)	1.119 (0.856, 1.463)	2.315 (1.873, 2.86)
S3A to S3B	0.519 (0.452, 0.597)	0.929 (0.708, 1.22)	0.801 (0.564, 1.137)	0.775 (0.59, 1.017)
S3B to S4	0.151 (0.124, 0.183)	1.24 (0.876, 1.756)	0.947 (0.591, 1.517)	1.378 (0.928, 2.048)
S3B to TT	0.006 (0, 0.169)	1.516 (0.279, 8.239)	0.364 (0.011, 12.50)	0.025 (0, 77.97)
S4 to TT	0.027 (0.014, 0.052)	1.337 (0.43, 4.152)	0.89 (0.241, 3.295)	0.082 (0.018, 0.369)

AIC: 8174.441.

We examined the effect on scarring and TT transition of the household variables. The relationships with scarring transitions were largely in the early stages, from S0 to S1, and S1 to S2 (Supplementary Table 3).

Our final multivariate parsimonious model is shown in Table 4. The scarring transition rate from S0 to S1 and S1 to S2 was related to increasing age, increasing baseline CPTF, and independently to household ownership of a telephone. For parsimony, the relative rates were aggregated (which were small) where the model suggested. Relative rates of 1.00 show where the model omitted the covariate.

**Table 4:** Multivariate model of scarring transition rates

	Transition rate (per year)	Age (relative rate per decade)	Relative rate: second time period	Relative rate, 5–10% TF prevalence	Relative rate, > 10% TF prevalence	Relative rate: telephone	Relative rate: latrine
S0 to S1	0.047 (0.041, 0.053)	1.29 (1.24, 1.33)	11.33 (9.19, 1.97)	1.26 (1.13, 1.41)	1.48 (1.28, 1.70)	0.81 (0.74, 0.89)	1.00
S1 to S2	0.117 (0.105, 0.130)	1.29 (1.24, 1.33)	1.43 (1.17, 1.75)	1.26 (1.13, 1.41)	1.48 (1.28, 1.70)	0.81 (0.74, 0.89)	0.77 (0.63–0.94)
S2 to S3A	0.181 (0.162, 0.202)	1.00	2.27 (1.83–2.80)	1.00	1.00	0.81 (0.74, 0.89)	1.00
S3A to S3B	0.443 (0.37, 0.506)	1.04 (1.17, 1.10)	1.00	1.00	1.00	1.00	1.00
S3B to S4	0.149 (0.125, 0.177)	1.04 (1.17, 1.10)	1.00	1.00	1.00	1.00	1.00
S3B to TT	0.008 (0.004, 0.020)	1.00	0.06 (0.02–0.19)	1.00	1.00	1.00	1.00
S4 to TT	0.026 (0.015, 0.046)	1.00	0.06 (0.02–0.19)	1.00	1.00	1.00	1.00

AIC: 7847.589. The model includes baseline transition rate per year, the relative rate per decade of age, adjustment for the second time period (between second and third visits), baseline community TF prevalence categories, for telephone usage and latrine usage.

Using the rates from the multivariate model, we computed the expected proportion of women without scarring (S0) who progressed further per year. We assumed an age of 35 and the baseline mean level of the other parameters. For communities with TF levels below 5%, this proportion is approximately 1.33% (95% CI 1.09–1.64%) for the first follow-up period, and 14.0% (95% CI 13.0–15.2%) for the second follow-up period. For communities over 10% TF, this proportion is approximately 1.95% (95% CI 1.55–2.43%) in the first follow-up period, and 20.0% (95% CI 17.7–22.5%) in the second follow-up period.

We compared flow rates estimated for the first follow-up period between individuals with complete follow-up (all three visits) and individuals missing the final visit. We found no evidence that the flow rates differed (P > 0.05 for all seven estimates, estimates not shown). We also compared flow rates estimated from the initial and final visits, comparing individuals with complete follow-up with individuals missing the middle visit. We found evidence that the flow rate for transitioning from S0 to S1 differed; the relative rate for this group was estimated to be 0.834 (95% CI 0.70–0.99). All other flow rates exhibited no compelling evidence of a difference (P > 0.05 for all six).

Model adequacy was assessed as follows. First, we fit a model in which rates were permitted to be different by a constant factor before and after the first follow-up visit (year 3). We found no compelling evidence of nonstationarity (P = 0.32, likelihood ratio X^2^, 1 degree of freedom). Second, we conducted sensitivity analyses in which we removed the possibility of progression directly from stage 3B to TT, and also added the possibility of progression from 3A directly to TT; these model structural changes led to only minor differences in estimated rates (results not shown). Finally, we conducted an assessment of the Markov assumption for progression from stage S1 in the second follow-up period, contrasting individuals who began in stage S1 at baseline with those who had just progressed from S0 to S1 in the first time period; we obtained an estimated odds ratio for progression of 1∙1 (95% CI 0.60–2.04; P = 0.80, Fisher’s Exact Test). Finally, the relative McFadden R^2^ estimated for the multivariate model was 0.763.

## Discussion/interpretation

We modeled a yearly rate of progression for each state, and found a transition rate of incident scarring (from S0 to S1) of 4.7%, with adjustment for the other factors. The progression of S1 to S2, S2 to S3A, and S3B to S4 ranged from 11.7 to 18.1% per year; the transition from S3A to S3B was much higher at 44.3%. This was unexpected and we explored possible reasons for this. It is not a function of a very tight transition, as S3A ranges from 30 to < 50% of the eyelid with scarring and S3B ranges from 50 to 90%. The raw data clearly indicated that a sizeable proportion of the baseline population did transition out of S3A, and the data suggest little difference in this rate between the two observation periods—if anything the rate was somewhat lower in the second observation period. There were no other variables in this data set that were uniquely associated with this transition, leaving us unable to account for the high rate.

Older age is an important covariate in the transition of scarring from one state to another. This finding is not surprising, particularly in the transition from S0 to S1, and S1 to S2. Trachomatous scarring is the result of repeated infections with *C. trachomatis* and increasing age among those with S0 and S1 has afforded more opportunity to acquire more infections. Age is less important for transitions from S2 onward. These associations of older age and scarring progression may reflect that within each scarring category, older women may be closer to the upper level of severity and thus more likely to transition to the next level at the next follow-up. Older age is not a significant factor for the transition to TT. This suggests that once an eyelid achieves a grade of S3B or S4, regardless of age, there are other factors that determine the chance of developing TT.

The importance of exposure to trachoma in the early transitions is supported by the finding that CPTF is a significant covariate only early in the scarring process. Previous studies also suggest that as the prevalence of TF in a district declines, the incidence of new scarring also declines^9^ but the progression rates are largely unchanged^11^. In a cross-sectional survey, the odds of any scarring was significant when the community TF prevalence was 10% or higher^12^ This finding is important, as it suggests that if communities can fall below a sustained 5% prevalence of TF, new scarring is less likely to develop. The transition from S0 to higher scar stages in the first observation period in those in communities with TF < 5% was 1∙3% compared to almost 2% in communities with TF > 10%. Even with a low district TF prevalence there is heterogeneity in the communities^13^ with some at greater increased risk of incident scarring. The data also suggest that if scarring at the level of S2 or greater is present, it will likely progress in severity regardless of low or absent community prevalence of TF. Thus, controlling incident scarring is a good way to lead to eventual elimination of TT as a public health problem. If there is still a sizeable component of the population with scarring at least at S2 severity, there will be continued progression to TT. However, at some point after elimination of active trachoma, especially if there are only a few hotspot villages, the risk of transition to TT will fall below the 2/1000 population range. In future work, we intend to use these data to model timelines to TT depending on age and community TF levels projected to district level.

The socioeconomic status markers at household level have all been associated with active trachoma in previous surveys, and so it is not surprising they were associated with the transition from S0 to S1 and S1 to S2 in univariate analyses. After adjustment for TF, most of these variables were not significantly associated with scarring transition. Household latrine and telephones were more proximal to the cases and may indicate household level risk of TF, whereas CPTF was assessed at community level.

The transition rate from S0 to S1 was significantly higher in the second observation period compared to the first observation period. During the first observation period any community with CPTF > 5% s received annual MDA during the first 3.5 years of follow-up, compared with no MDA in the second follow-up of 4.5 years. Supplementary Table 1 suggests a very rapid return to high levels of TF without MDA, and return of transmission. Thus, the scarring transition rate would be expected to accelerate in such conditions, and that is what we observed. The MDAs were interrupted by COVID and did not take place until after our last follow-up. The transition rate for the second observation period in the model was set to an average of the baseline TF rate, although ideally a CPTF in the midpoint of the second observation period would have been reflective of the large increase in TF rates and thus higher transition rates from S0.

There are limitations to our study. We enrolled the right eye only of participants at baseline, and for follow-up through over the 8 years. It is not clear why there should be a difference in progression between the two eyes, but we cannot rule this out.

We enrolled women only. This was driven by the original cross-sectional survey. Men have a lower risk of TT and recent data suggest that they do not progress as quickly as women do^12^ Thus, our estimates of incidence and progression to TT can only be applied to women, understanding that they carry the majority of the TT burden and will be the majority of cases once the country has eliminated active trachoma.

We do not have the date at which TT developed, only the observation at the study visits. It is possible that TT could have developed earlier in the process, say at level S3A. We calculated a low probability of transition from S3B to TT (0.8%/year) compared to S4 to TT (2.6%/year), suggesting it is not unreasonable to assume an even lower rate of direct transition from lower levels of scarring to TT. The effect would be to underestimate the transition from grades below S3B to TT, and to overestimate the transition to TT from S3B and S4. However, the calculated transitions support TT associated with S3B and S4, even with some misclassification.

We experienced loss to follow-up, more often in younger women with more severe scarring at baseline. We do not know if the chance of progression for these women would be different within each state of scarring, but we could postulate it might be lower because they were younger. There were also very modest SES differences, mostly 1% or less in percent. There was no significant difference in CPTF.

We made assumptions when choosing our model. First, scarring does not regress, a reasonable assumption given the biology of the scarring process and its replacement of normal conjunctiva. Second, we assumed a Markovian structure in which the probability of progression depends only on the current scar state (given the covariates); we failed to find evidence to disconfirm this assumption. Third, we omitted the possibility of direct transitions in which scar stages are skipped (e.g., from S1 to S3), thereby assuming a gradual, as opposed to saltatory, worsening of scarring over time.

There are considerable strengths to our model. We have a large sample size, followed for 8 years, which allows time to model the transitions. As far as we know this is the only longitudinal dataset on scarring and will lend itself to projecting a time course for reducing the development of TT under different scenarios. The scarring data were based on multiple graders’ image scores, and thus were likely more robust than a single grader in the field. It also permits data checking to be certain the same person was followed at each visit, so we are confident in the progressions observed.

Our model represents the first effort to characterize yearly transitions through no scarring to trichiasis, adjusting for modifying factors. Despite limitations noted above, the modifying factors are consistent with other studies for factors associated with trachomatous scarring. Our future plans are to build theoretical model districts that use our transition probabilities to construct an estimate of time to TT dependent on baseline scarring rates, age structure, and TF prevalence heterogeneity. Such models could provide estimates of when the district, having eliminated active trachoma, is likely to have a prevalence of incident TT below 2/1000 population ages fifteen and older.

### Supplementary Information

Supplementary Information.

## Data Availability

Individual de-identified data on scarring and trichiasis over time, and community and household risk factors at baseline, will be made available to researchers with a signed data access agreement upon publication. Data will be available online via the COR-NTD Research Dataverse: [DOI] will be sent on acceptance. https://dataverse.unc.edu/dataverse/COR-NTD.
