# Supplementary material for: Model of yearly transition to severe trachomatous scarring and trichiasis in a cohort of women in Kongwa Tanzania
Source: Sci Rep. 2024 Jul 19;14:16654. doi: 10.1038/s41598-024-67245-w (PMC11271489; doi:10.1038/s41598-024-67245-w)
Supplement: Supplementary file 1 — Supplementary Information. [file 41598_2024_67245_MOESM1_ESM.docx]

Supplemental Tables and Figure

**Table S1: A.Community TF prevalence at baseline and in 2015 for 48 communities and in 2018 for 16 of the 48 communities.**

| Village TF prevalence | Survey year | | |
| --- | --- | --- | --- |
|  | 2013  N=48 | 2015  N=48 | 2018  N=16 |
| <5% | 56.2 | 77.1 | 12.5 |
| 5% to <10% | 29.2 | 18.7 | 43.7 |
| ≥10% | 14.6 | 4.2 | 43.8 |
| Average TF prevalence (mean (SD)) | 5.2 (3.9) | 3.4 (2.7) | 10.4 (6.4) |

B. Community TF prevalence restricted to the 16 villages with TF data available in 2018.

| Village TF prevalence | Survey year | | |
| --- | --- | --- | --- |
|  | 2013  N=16 | 2015  N=16 | 2018  N=16 |
| <5% | 81.3 | 75.0 | 12.5 |
| 5% to <10% | 12.5 | 25.0 | 43.7 |
| ≥10% | 6.2 | 0.0 | 43.8 |
| Average TF prevalence (mean (SD)) | 4.0 (3.6) | 2.9 (2.5) | 10.4 (6.4) |

**Table S2: Comparison of those followed-up from baseline to first follow-up only, and those followed from baseline to second follow-up only with those followed-up at both time points.**

| Baseline Characteristics | Not followed after baseline  (GROUP 1) | First follow-up only  (GROUP 2) | Second follow-up only  (GROUP 3) | First and second follow-up  (GROUP 4) | All | P-value  Comparing group 4 and group 2 | P-value  Comparing group 4 and group 3 |
| --- | --- | --- | --- | --- | --- | --- | --- |
| N | 1018 | 614 | 553 | 1830 | 4015 |  |  |
| Age in years |  |  |  |  |  |  |  |
| Mean (SD) | 32.9 (17.3) | 33.9 (16.7) | 35.7 (16.3) | 36.2 (15.0) | 34.9 (16.1) | **0.003** | 0.49 |
| Median | 27 | 29 | 32 | 33 | 31 |  |  |
| Min- Max | 15- 95 | 15 - 94 | 15 - 84 | 15 - 86 | 15 - 95 |  |  |
| TF village prevalence |  |  |  |  |  |  |  |
| Mean (SD) | 5.3 (4.0) | 5.6 (3.8) | 5.3 (4.0) | 5.4 (3.8) | 5.4 (3.8) | 0.36 | 0.57 |
| Median | 4.2 | 4.9 | 4.8 | 4.8 | 4.8 |  |  |
| Min- Max | 0 – 15.7 | 0 – 15.7 | 0 – 15.7 | 0 – 15.7 | 0 – 15.7 |  |  |
| Years of formal education |  |  |  |  |  |  |  |
| Mean (SD) | 3.7 (3.7) | 4.0 (3.6) | 3.6 (3.5) | 3.9 | 3.8 (3.5) | 0.47 | 0.18 |
| Median | 4.0 | 7.0 | 4.0 | 5.0 | 4.0 |  |  |
| Min- Max | 0 - 14 | (0 – 14) | (0 -12) | (0 – 14) | 0 - 14 |  |  |
| Family owns a bicycle (n (%)) | 424 (41.9) | 276 (45.3) | 253 (46.0) | 881 (48.4) | 1834 (45.9) | 0.19 | 0.33 |
| Family owns a phone (n (%)) | 459 (45.3) | 290 (47.5) | 241 (43.9) | 824 (45.3) | 1814 (45.4) | 0.33 | 0.58 |
| House has a mud roof (n (%)) | 161 (15.9) | 75 (12.3) | 77 (14.0) | 188 (10.3) | 501 (12.5) | 0.18 | **0.02** |
| House has a latrine (n (%)) | 787 (77.8) | 484 (79.9) | 440 (80.0) | 1479 (81.3) | 3190 (80.0) | 0.45 | 0.51 |
|  |  |  |  |  |  |  |  |
| TS Grade n (%) |  |  |  |  |  |  |  |
| S0 | 637 (62.6) | 461 (75.1) | 329 (59.5) | 1283 (70.1) | 2710 (67.5) | 0.15* | **<0.002*** |
| S1 | 203 (19.9) | 59 (9.6) | 116 (21.0) | 234 (12.8) | 612 (15.2) |  |  |
| S2 | 95 (9.3) | 57 (9.3) | 65 (11.8) | 210 (11.5) | 427 (10.6) |  |  |
| S3A-S3B | 63 (6.2) | 24 (3.9) | 33 (6.0) | 63 (3.4) | 183 (4.6) |  |  |
| S4 | 20 (2.0) | 13 (2.1) | 10 (1.8) | 40 (2.2) | 83 (2.1) |  |  |

*Test for trend

**Table S3: time dependent models for transition between scarring severity levels and between scarring and trachomatous trichiasis for household variables: Relative rate and 95% confidence intervals for adjusted for age, community prevalence of TF, and time period*.**

|  | Relative rate: Latrine | Relative rate: Bicycles | Relative rate: Time to Water | Relative rate : Education | Relative rate: Mud Roof | Relative rate: Telephone |
| --- | --- | --- | --- | --- | --- | --- |
| S0 to S1 | 0.937  (0.787-1.12) | 0.967  (0.837-1.12) | 1.05  (0.968-1.15) | **0.784**  **(0.64-0.96** | 1.08  (0.87-1.34) | **0.861**  **(0.741-0.999)** |
| S1 to S2 | **0.790**  **(0.640- 0.974)** | 0.953  (0.790-1.15) | 1.03  (0.916-1.15) | 0.879  (0.673, 1.15) | 1.18  (0.920, 1.51) | 0.873  (0.718, 1.06) |
| S2 to S3A | 0.952  (0.750, 1.21) | 0.911 (0.738, 1.13) | 1.05  (0.926, 1.18) | 1.02  (0.759, 1.37) | **0.662**  **(0.496, 0.884)** | 0.811  (0.650, 1.01) |
| S3A to S3B | 0.933  (0.684, 1.27) | 0.919 (0.704, 1.20) | 1.02  (0.866, 1.20) | 0.915  (0.619, 1.35) | 0.853  (0.568, 1.28) | 1.02  (0.759, 1.36) |
| S3B to S4 | 0.930  (0.628, 1.38) | 0.943 (0.664, 1.34) | 0.939 (0.770, 1.15) | 0.751  (0.439, 1.29) | **1.79**  **(1.14, 2.81)** | 1.34  (0.901, 1.99) |
| S3B to TT** | 0.0313  (0.002, 0.470) | 173 (0.002, 1.31 ^7) | 0.529 (0.124, 2.27) | 0.00047 (3.61 ×10^-7^, 0.602) | 0.0186 (6.20 ×10^{-9}, 55900) | 45.2  (1.52, 1340) |
| S4 to TT | 1.18  (0.477, 2.93) | 0.733 (0.255, 2.10) | 1.16  (0.705, 1.91) | 1.00  (0.267, 3.75) | 1.16  (0.486, 2.75) | 0.0014 (2.49 ×10^{-12}, 7.71×10^{5}) |
|  |  |  |  |  |  |  |
|  |  |  |  |  |  |  |
| AIC |  |  |  |  |  |  |

*Each model includes a baseline transition rate, a relative rate adjustment for CPTF, and a relative rate adjustment for the second follow-up period (results not shown). The rows correspond to a model with a different estimated relative rate for each flow rate.

**Estimates for this row are very unstable due to small numbers and are shown for completeness.

**Supplemental Figure 1: Diagram of progression of scarring, allowing scarring to progress to trichiasis from the most severe levels of scarring.**
